# Internal exposure to neutron-activated ^56^Mn dioxide powder in Wistar rats—Part 2: pathological effects

**DOI:** 10.1007/s00411-016-0676-z

**Published:** 2017-02-08

**Authors:** Kazuko Shichijo, Nariaki Fujimoto, Darkhan Uzbekov, Ynkar Kairkhanova, Aisulu Saimova, Nailya Chaizhunusova, Nurlan Sayakenov, Dariya Shabdarbaeva, Nurlan Aukenov, Almas Azimkhanov, Alexander Kolbayenkov, Zhanna Mussazhanova, Daisuke Niino, Masahiro Nakashima, Kassym Zhumadilov, Valeriy Stepanenko, Masao Tomonaga, Tolebay Rakhypbekov, Masaharu Hoshi

**Affiliations:** 1 Nagasaki University, 1-12-4, Sakamoto, Nagasaki, 852-8523 Japan; 2 Hiroshima University, 1-2-3, Kasumi, Minami-ku, Hiroshima,, 734-8553 Japan; 3 Semey State Medical University, Republic of Kazakhstan, Abay Str., 103, Semey, 071400 Kazakhstan; 4 National Nuclear Center of the Republic of Kazakhstan, Krasnoarmeyskaya Str., 2, Build 54 B, Kurchatov, 071100 Kazakhstan; 5 L.N. Gumilyov Eurasian National University, Munaitpasova Str.,13, Astana, 010008 Kazakhstan; 6 A. Tsyb Medical Radiological Research Center, National Medical Research Radiological Center, Ministry of Health of Russian Federation, Koroleva Str. 4, Obninsk, Kaluga region 249036 Russia

**Keywords:** Manganese-56, Internal radiation exposure, Lung, Rats, A-bombing

## Abstract

To fully understand the radiation effects of the atomic bombing of Hiroshima and Nagasaki among the survivors, radiation from neutron-induced radioisotopes in soil and other materials should be considered in addition to the initial radiation directly received from the bombs. This might be important for evaluating the radiation risks to the people who moved to these cities soon after the detonations and probably inhaled activated radioactive “dust.” Manganese-56 is known to be one of the dominant radioisotopes produced in soil by neutrons. Due to its short physical half-life, ^56^Mn emits residual radiation during the first hours after explosion. Hence, the biological effects of internal exposure of Wistar rats to ^56^Mn were investigated in the present study. MnO_2_ powder was activated by a neutron beam to produce radioactive ^56^Mn. Rats were divided into four groups: those exposed to ^56^Mn, to non-radioactive Mn, to ^60^Co γ rays (2 Gy, whole body), and those not exposed to any additional radiation (control). On days 3, 14, and 60 after exposure, the animals were killed and major organs were dissected and subjected to histopathological analysis. As described in more detail by an accompanying publication, the highest internal radiation dose was observed in the digestive system of the rats, followed by the lungs. It was found that the number of mitotic cells increased in the small intestine on day 3 after ^56^Mn and ^60^Co exposure, and this change persisted only in ^56^Mn-exposed animals. Lung tissue was severely damaged only by exposure to ^56^Mn, despite a rather low radiation dose (less than 0.1 Gy). These data suggest that internal exposure to ^56^Mn has a significant biological impact on the lungs and small intestine.

## Introduction

After the atomic bombing of Hiroshima and Nagasaki, Japan, initial radiation directly produced during or shortly after the explosions and residual radiation contributed towards a radiation exposure of the survivors (Imanaka et al. 2008). There are two sources of residual radiation: (1) neutron-activated radioisotopes from materials on the ground and (2) radioactive fallout containing fission products and residual fissile materials from the bombs. Understanding the former is particularly important for evaluating the risks to those people who moved to these cities soon after the detonations and might have inhaled radioactive dust (Kerr et al. 2013, 2015; Imanaka et al. 2008). Such individuals were reported to suffer from various syndromes similar to acute radiation effects (Imanaka et al. 2012). The neutron-induced radioisotopes include ^24^Na, ^28^Al, ^31^Si, ^32^P, ^32^Cl, ^42^K, ^45^Ca, ^48^Sc, and ^56^Mn, among others (Tanaka et al. 2008). In terms of radiation exposure, ^56^Mn (physical half-life: 2.58 h), which emits both β particles and γ rays, is one of the most important radioisotopes produced after the atomic bomb explosion in Hiroshima.

To understand the radiation effect of ^56^Mn, neutron-activated ^56^MnO_2_ powder was sprayed over rats, and its biological effects were evaluated. The highest doses of internal irradiation were recorded in the digestive system, followed by the lungs (Stepanenko et al. 2016a, b). Here the results of histological examinations in rats exposed to ^56^Mn are reported.

## Materials and methods

### Animals

Five-month-old male Wistar rats were obtained from Karaganda State Medical University, Kazakhstan. They were maintained with free access to basal diet and tap water. In Experiment 1, rats were divided into four groups, with six rats for the ^56^Mn group and three rats per group for the Mn, ^60^Co, and control groups. The ^56^Mn and Mn groups were exposed to ^56^MnO_2_ and non-radioactive MnO_2_, respectively. The ^60^Co group received 2 Gy of external ^60^Co γ-ray irradiation. Three rats of the ^56^Mn group were killed for dosimetry 3.5–4 h after the exposure. One rat from each group was killed on days 3, 14, and 60. In Experiment 2, the exposure was repeated with 12 rats in the ^56^Mn group and nine rats each in the Mn, ^60^Co, and control groups. Three rats of the ^56^Mn group were killed for dosimetry 3.5–4 h after the exposure. Then, three rats from each group were killed and examined on days 3, 14, and 60 after the irradiation (Table 1).

**Table 1:** Experiment groups

Exposure	Dose (whole body)	Dosimetry	Number of rats			Initial body weights (g, mean ± SE)
			Day 3	Day 14	Day 60	
Experiment 1						
^56^Mn	0.15 Gy	3	1	1	1	217 ± 5.8
Mn	0 Gy	–	1	1	1	237 ± 8.6
^60^Co	2 Gy	–	1	1	1	198 ± 9.0
Control	0 Gy	–	1	1	1	190 ± 23.1
Experiment 2						
^56^Mn	0.02 Gy	3	3	3	3	202 ± 6.7
Mn	0 Gy	–	3	3	3	202 ± 5.8
^60^Co	2 Gy	–	3	3	3	207 ± 11.0
Control	0 Gy	–	3	3	3	202 ± 9.6

### Irradiation and dosimetry of each organ of rats

Details of irradiation using ^56^Mn and the corresponding internal dose estimation have been described in Stepanenko et al. 2016a and Stepanenko et al. 2016b. In brief, ^56^MnO_2_ was obtained by neutron activation of 100 mg of MnO_2_ powder using the Baikal-1 nuclear reactor at Kurchatov, Kazakhstan. A thermal neutron fluence of 4 × 10^14^ n/cm^2^ was applied to produce 2.74 × 10^8^ Bq of radioactivity of ^56^Mn. The activated MnO_2_ powder was sprayed into sealed boxes containing six rats per box (one box was used for Experiment 1, and two boxes were used for Experiment 2). In Experiment 1, the exposure box was equipped with air filters only, while an additional forced ventilation system was installed to improve animal welfare in the boxes in Experiment 2. The same total activity of ^56^Mn equal to 2.74 × 10^8^ Bq was used for irradiation in both experiments. The specific activity of ^56^Mn powder was the same (2.74 × 10^9^ Bq/g) as well. After 1 h, rats were removed from the exposure box(es) into fresh cages, cooled down for 2.5–3 h. Then, three of the animals were killed by intraperitoneal injection of an excessive dose of pentobarbital. A piece of each organ was dissected, weighted and put into a vial. The radioactivity of ^56^Mn in samples of each organ was measured with a gamma spectrometer. The absorbed fractions from beta and gamma irradiation of each organ as well as the whole body were calculated based on the Monte Carlo code (version MCNP-4C) and the mathematical phantom of a rat. Assessment of internal radiation doses was performed by measuring of ^56^Mn activity in each organ and calculating the absorbed fractions of internal exposure to photons and electrons. Details of the dosimetry are described in an accompanying paper (Stepanenko et al. 2016a).

Whole body γ-ray irradiation of 2 Gy was performed at a dose rate of 2.6 Gy/min using a Teragam K-2 unit (UJP Praha, Praha-Zbraslav, Czech Republic).

### Pathology

The liver, heart, kidney, trachea, lungs, tongue, esophagus, stomach, small intestine, eyes, and skin were dissected, fixed in 10 % formalin, and embedded in paraffin. Sections of 4 μm thickness were prepared and stained with hematoxylin and eosin (HE). For scoring of mitotic cells in the intestinal crypt, good longitudinal sections of the crypt that aligned with other crypts and contained crypt lumen were selected. At least 30 sections per rat were examined under a light microscope, as described previously (Matsuu et al. 2000; Matsuu-Matsuyama et al. 2010). For pathological examination of the lung tissue, the grades of hemorrhage, emphysema, inflammation (inflammatory cell population), expression of lymphoid follicle, and alveolar wall hypertrophy were scored from “−” to “+++” (Shichijo et al. 2000, 2007).

### Statistical analysis

All values were expressed as the mean ± standard error (SE). Mann–Whitney’s *U* test was applied to evaluate the statistical significance of difference between groups.

## Results

### Radiation doses due to ^56^Mn exposure

The radiation dose received from ^56^Mn varied among different organs (Table 2). Although the initial activities of neutron-activated MnO_2_ powder were similar in Experiments 1 and 2, the radiation doses of each organ received in Experiment 2 were substantially lower than those received in Experiment 1, i.e., the small intestine received 1330 mGy in Experiment 1 while 150 mGy in Experiment 2. However, the distribution of dose values between tissues was similar in the two experiments, being very high in the digestive system, followed by the lungs and skin. Details are given in Stepanenko et al. (2016a).

**Table 2:** Specific activity of ^56^Mn, A0, and accumulated doses of internal irradiation, D, in different organs of experimental rats

Organs	Experiment 1^*, #^		Experiment 2^*, #^	
	A_0_ (kBq/g)	D (mGy)	A_0_ (kBq/g)	D (mGy)
Liver	4.1 ± 0.35	15 ± 1.4	0.48 ± 0.06	1.7 ± 0.2
Heart	5.5 ± 0.6	16 ± 2.1	0.47 ± 0.05	1.3 ± 0.2
Kidney	5.8 ± 0.48	13 ± 2.0	0.1 ± 0.013	0.3 ± 0.05
Trachea	14 ± 0.75	14 ± 2.0	3 ± 0.3	7.3 ± 0.9
Lung	72 ± 9.3	100 ± 14	23 ± 2.5	30 ± 4.0
Tongue	45 ± 5.4	69 ± 11	7.1 ± 1.2	11 ± 2.0
Esophagus	26 ± 3.6	50 ± 9.0	3.6 ± 0.37	7.1 ± 0.9
Stomach	150 ± 16	240 ± 30	69 ± 7.2	110 ± 13
Small intestine	810 ± 93	1330 ± 170	89 ± 9.3	150 ± 17
Eyes	13 ± 1.7	21 ± 3.0	17 ± 2.4	26 ± 4.0
Skin	41 ± 4.9	76 ± 10	39 ± 5.5	73 ± 13
Whole body	83 ± 11	150 ± 25	23 ± 3.4	41 ± 7.5

A forced ventilation system was used only in Experiment 2 during the exposure

*Cited from Stepanenko et al. (2016a, b)

^#^Total activity of sprayed ^56^MnO_2_ powder was 2.74  ×  10^8^ Bq in both Experiments

### Pathological findings

**Fig. 1:**
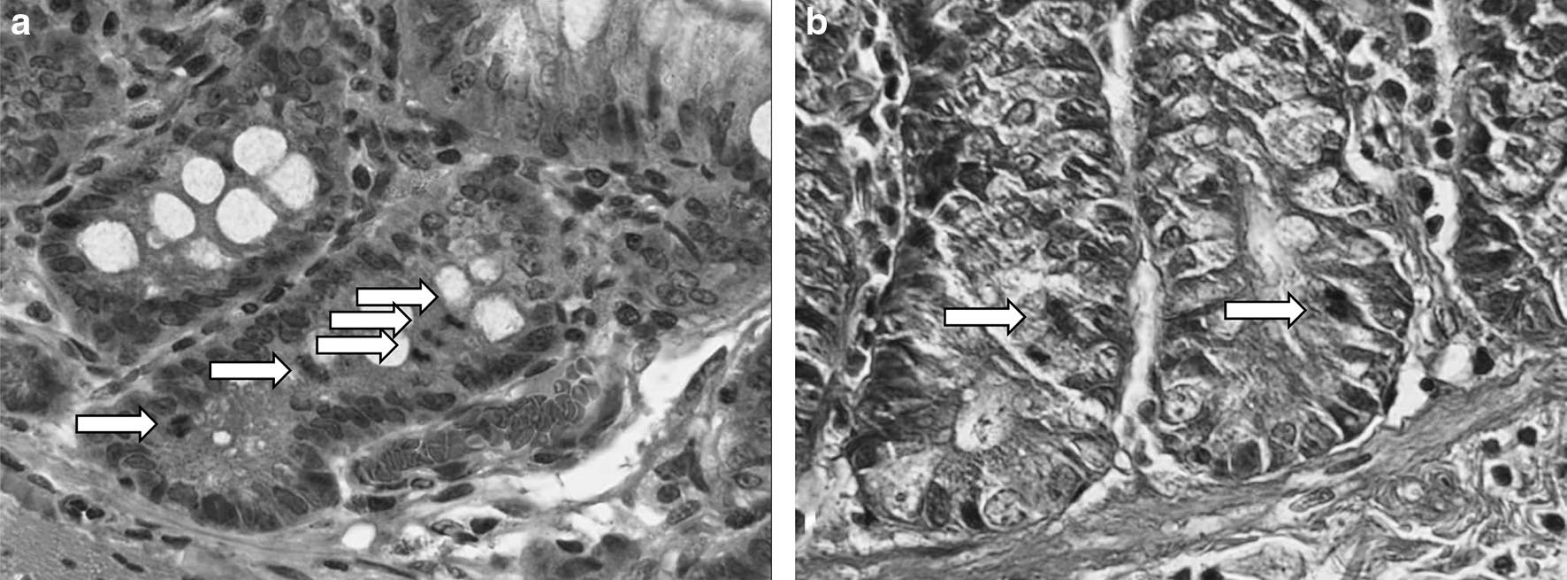
Small intestine of rats 60 days after exposure. A number of mitotic cells (*arrows*) per crypt were noted in the ^56^Mn group (**a**); the number in the ^60^Co group (**b**) was similar to that in the control group. HE staining, original magnification 100×

The rats appeared to remain healthy during the study, in both experiments; no deaths were recorded. Compared to the control groups, there were no macropathological changes in the ^56^Mn, Mn, or ^60^Co groups. Exposure-related histological changes were noted only in the small intestines and lungs. The number of mitotic cells per crypt in the small intestine is summarized in Table 3 (see also Fig. 1). The number increased in both the ^56^Mn and ^60^Co groups on day 3 after the exposure. While this number returned to the control level by day 14 in the ^60^Co group, it remained high on day 14 in the ^56^Mn group of Experiment 2 and high on day 60 in the ^56^Mn group of Experiment 1 as well.

**Table 3:** Number of mitotic cells per crypt in rat small intestine in ^56^Mn, Mn, ^60^Co and control groups

	Day 3	Day 14	Day 60
Experiment 1			
^56^Mn	1.81 ± 0.26^*^	1.14 ± 0.14	2.83 ± 0.24^*,#^
Mn	1.07 ± 0.20	0.98 ± 0.13	1.71 ± 0.24
^60^Co	2.19 ± 0.25^*^	0.89 ± 0.11	1.38 ± 0.18
Control	0.95 ± 0.18	1.06 ± 0.22	1.32 ± 0.20
Experiment 2			
^56^Mn	1.73 ± 0.12^*^	1.31 ± 0.12^*,#^	0.96 ± 0.10
Mn	0.71 ± 0.08	0.63 ± 0.06	0.73 ± 0.08
^60^Co	1.43 ± 0.14^*^	0.66 ± 0.08	0.75 ± 0.09
Control	0.94 ± 0.20	0.76 ± 0.10	0.78 ± 0.10

Mean ± SE

* *p* < 0.05 vs. Control, ^#^ *p* < 0.05 vs. Co-60

**Fig. 2:**
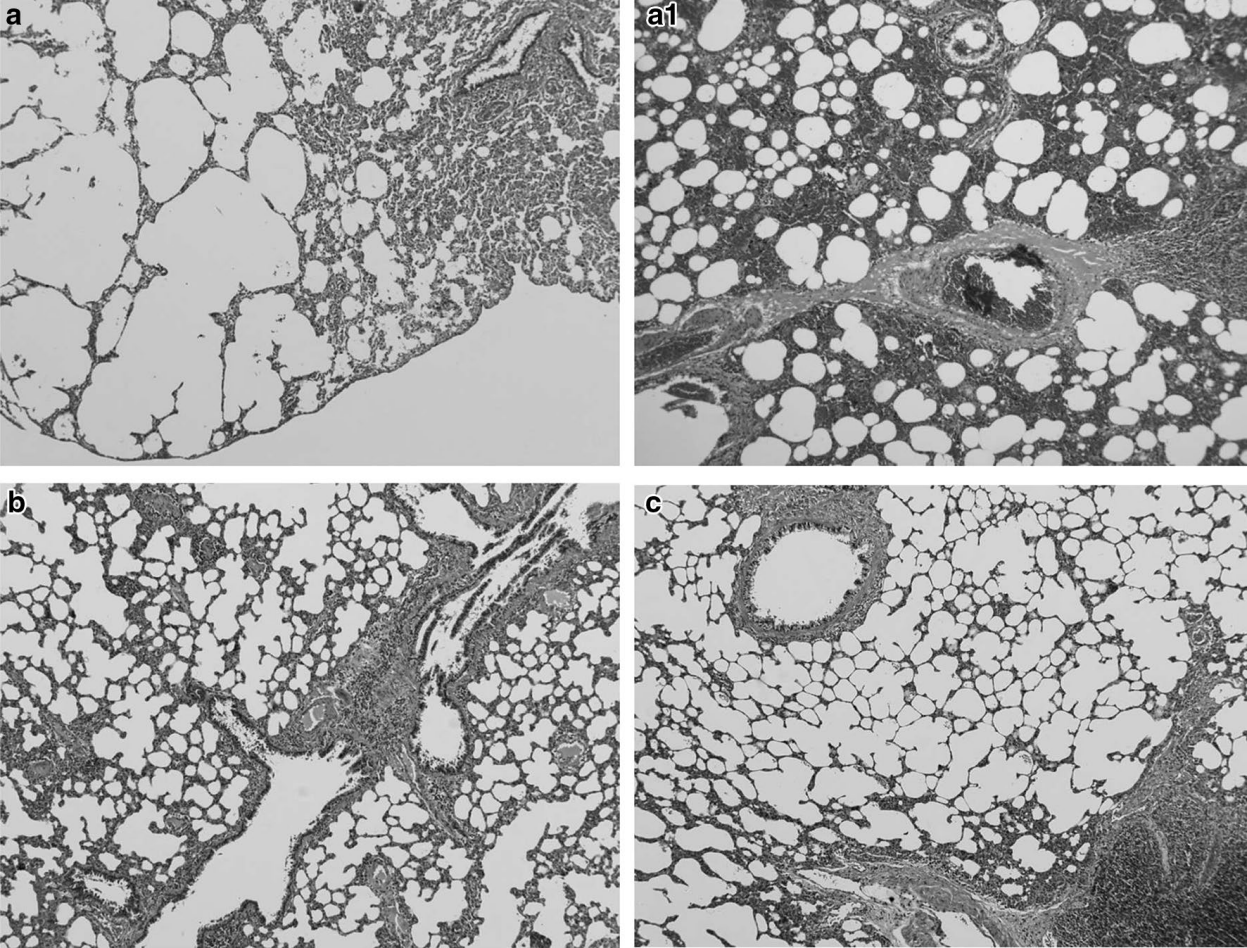
Lung of rats 3 days after exposure. Severe emphysema (**a**), and hemorrhage (**a1**) were observed in the ^56^Mn group. No pathological changes were observed in the Mn group (**b**). Control group (**c**). HE staining, original magnification ×10

Table 4 summarizes the histological changes in the lungs. In Experiment 1, damage to the lung tissue, including hemorrhage, emphysema, and inflammation, was evident on days 3 and 14 in the ^56^Mn group (Fig. 2). ^60^Co exposure, on the other hand, did not cause any significant changes in the lung tissue. The effect of ^56^Mn on the lungs in Experiment 2 was not as pronounced as that in Experiment 1, but hemorrhage and emphysema were still noted in the ^56^Mn group.

**Table 4:** Histological findings in the lung in rats exposed to ^56^Mn, Mn, ^60^Co and control groups

		Day3	Day14	Day60
Experiment 1				
^56^Mn	Hemorrhage	+++	++	+
	Emphysema	++	+	+
	Inflammation	+++^a^	++	+
Mn	Hemorrhage		+	+
	Emphysema	+		
	Inflammation	+	+	+
^60^Co	Hemorrhage			
	Emphysema			
	Inflammation		+	+
Control	Hemorrhage	+	+	
	Emphysema			
	Inflammation	+	+	
Experiment 2				
^56^Mn	Hemorrhage	(+)^b^	(+)	
	Emphysema		(+)	
	Inflammation	(+~++)	(++)	(+)
Mn	Hemorrhage			
	Emphysema			
	Inflammation	(+)	(+)	(+~++)
^60^Co	Hemorrhage			
	Emphysema			(+)
	Inflammation	(+~++)	(+)	(+)
Control	Hemorrhage		(+)	
	Emphysema			
	Inflammation	(++)	(+~++)	(+)

^a^Pathological grades were scored from − to +++

^b^Incidence and pathological grades (in parenthesis)

^c^Occasional lymphoid follicles and alveolar wall hypertrophy were noted without significant differences among the exposed and control groups

## Discussion

In the present study, two independent experiments were performed with laboratory rats exposed to neutron-activated ^56^MnO_2_ powder. Although the absorbed doses of internal irradiation received from ^56^Mn were rather low in Experiment 2, the observed biological effects were consistent in both the experiments. The lungs were severely damaged by ^56^Mn, with histological changes, including hemorrhage and emphysema, being present 2 weeks after the exposure. In the small intestine, mitosis was enhanced for an extended period after exposure to ^56^Mn.

These results might demonstrate that, to understand the radiation effects among the survivors of the atomic bombing of Hiroshima and Nagasaki, it is important to include the effects of residual radiation in addition to those of the initial radiation directly received from the bombs. Residual radiation consists of neutron-induced radioisotopes on the ground and radioactive fallout from the bomb, known as “black rain” (Imanaka et al. 2008). In terms of radiation dose, among the neutron-induced radioisotopes, ^56^Mn is a dominant radioisotope produced during the atomic bomb explosion (Tanaka et al. 2008). Since ^56^Mn and the other neutron-activated radioisotopes were present in dust after the bombings, people might have inhaled these radioactive materials and been internally exposed to the radiation. It is important to note that individuals who returned early to Hiroshima and Nagasaki after the atomic bombing were reported to suffer from the symptoms of acute radiation effects (Imanaka et al. 2012). The present international study aimed to investigate the effects of exposure to major residual radioactive particles found in the dust after the atomic bomb explosion by carrying out an animal experiment on the exposure of ^56^Mn powder to Wistar rats. Although two experiments were performed using the same initial total and specific activity of powdered ^56^Mn, the absorbed doses due to incorporated ^56^Mn were substantially higher in Experiment 1 than in Experiment 2. Note, however, that the activity during the irradiation was not measured. Particle size of MnO_2_ powder was measured and a similar size distribution was found for both experiments ranging from 4 to 10 μm in diameter. The difference between the two experiments was in the ventilation system. In Experiment 2, a forced ventilation system was installed in addition to air filters in the exposure boxes. This may have affected the absorbed doses of internal radiation. Nevertheless, the doses received by the skin were similar in both experiments, which may suggest that the ^56^MnO_2_ powder was not taken up very well by either inhalation or ingestion in the rats in Experiment 2, probably due to the difference in ventilation. Note that although the highest radiation doses were observed in the digestive system of the animals in both experiments, this may not be the case in humans because rats ingest a lot of radioactive powder during grooming. The higher radiation doses found in lung tissue, on the other hand, represent an exposure route in humans who inhale dust containing radioactive particles.

It is well known that ionizing radiation increases the occurrence of apoptosis in the crypts of the small intestine, being observed within a period of 3–6 h in rodents (Becciolini et al. 1986; Potten and Grant 1998; Matsuu et al. 2010). The mitotic index, on the other hand, was reported to gradually increase and peaked at 3–4 days after the exposure (Potten and Grant 1998). This coincided with the present data showing increases in mitotic cell numbers on day 3 in both the ^56^Mn and ^60^Co groups. Interestingly, in Experiment 2, an increase in mitosis was still observed on day 14 after exposure to ^56^Mn, while it returned to the control level in the ^60^Co group, suggesting that the effects of internal radiation of ^56^Mn were more persistent. However, an increase in mitosis was not found on day 14 but on day 60 in Experiment 1. Further studies are needed to determine whether this effect was due to the difference in radiation dose.

It is also well known that radiation exposure of the lungs can induce “radiation pneumonitis” in laboratory animals, at lung doses above 8 Gy (Coggle et al. 1986; Ward et al. 1992). Studies in rats showed that a thorax irradiation of 20 Gy did not induce any short-term histological changes in the lungs, although the irradiated lungs developed focal exudative lesions after 2 months and then reparative fibrotic lesions by 6 months (Travis et al. 1977; Down 1986). Lung function was also damaged by irradiation with a single dose of 12 Gy (Eerde et al. 2001). These data are consistent with findings of the present pathological examination of the lung tissue in the ^60^Co group (2 Gy), which did not show any significant changes. In contrast to the external γ-radiation, the internal exposure to ^56^Mn induced pathological changes, including hemorrhage and emphysema, although the radiation doses were low: 100 mGy in Experiment 1 and 30 mGy in Experiment 2. The stronger biological effects of exposure to ^56^Mn may be due to its β emissions. Whether these initial pathological changes lead to any long-term lesions should be investigated in the future. Previous studies investigating the effects of inhaled radioactive plutonium, an α-emitter, showed that rats died with severe pulmonary edema at higher radiation doses, while radiation pneumonitis and emphysema developed at lower doses (Lundgren et al. 1981; Dagle and Sanders 1984; Scott et al. 1990). In the present study, occasional inflammations were observed in the lung in control group. This is probably because all of the animals were maintained in the conventional facility since the radiation exposure took place in the non-SPF (Specific-Pathogen Free) condition due to the technical limitation at the nuclear reactor.

Manganese is well known for its neurotoxicity (Crossgrove and Zheng 2004; O’Neal and Zheng 2015). In humans, Mn overexposure induces symptoms similar to those of Parkinson’s disease, although animal studies suggested that the mechanisms underlying toxicity are different (O’Neal and Zheng 2015). Manganese, particularly when administered by inhalation, may be toxic to the lung tissue, although results from epidemiological studies investigating the relationship between exposure to Mn and pulmonary diseases do not provide consistent results (Bergström 1977). Experimental studies have suggested that Mn exposure leads to a primary inflammatory reaction in the respiratory tract (Adkins et al. 1980; Camner et al. 1985). In the present study, there were no signs of toxicity by non-radioactive Mn, although a high dosage of MnO_2_ (100 mg) was sprayed into the exposure boxes. Since MnO_2_ powder was sprayed into the boxes without using any apparatus to agitate the powder or produce an aerosol, and because the exposure period was only 1 hour, the total amount of MnO_2_ inhaled by any rat would have been limited. Therefore, histological changes observed in the ^56^Mn group were probably the result of radiation exposure and not of chemical toxicity.

## Conclusion

This study investigated the effects of radiation exposure by ^56^MnO_2_ powder in male Wistar rats over 60 days. Although whole body radiation doses from ^56^Mn were relatively low, higher internal doses were noted in the small intestine and lungs, in addition to significant pathological changes that were more severe and prolonged than the effects of ^60^Co γ irradiation. These data may hint towards a potential risk of internal exposure to ^56^Mn, which might have existed in airborne dust after the atomic bomb explosions over Hiroshima and Nagasaki, Japan.
